# A transversal approach to predict gene product networks from ontology-based similarity

**DOI:** 10.1186/1471-2105-8-235

**Published:** 2007-07-02

**Authors:** Julie Chabalier, Jean Mosser, Anita Burgun

**Affiliations:** 1 E.A 3888, Modélisation Conceptuelle des Connaissances Biomédicales, Faculté de Médecine, Université de Rennes 1, IFR 140, 35043 Rennes Cedex, France; 2 CNRS UMR 6061 Génétique et Développement, Faculté de Médecine, Université de Rennes 1, IFR 140, 35043 Rennes Cedex, France; 3 OUEST-genopole^® ^transcriptomic platform, Faculté de Médecine, Université de Rennes 1, IFR 140, 35043 Rennes, France

## Abstract

**Background:**

Interpretation of transcriptomic data is usually made through a "standard" approach which consists in clustering the genes according to their expression patterns and exploiting Gene Ontology (GO) annotations within each expression cluster. This approach makes it difficult to underline functional relationships between gene products that belong to different expression clusters. To address this issue, we propose a transversal analysis that aims to predict functional networks based on a combination of GO processes and data expression.

**Results:**

The transversal approach presented in this paper consists in computing the semantic similarity between gene products in a Vector Space Model. Through a weighting scheme over the annotations, we take into account the representativity of the terms that annotate a gene product. Comparing annotation vectors results in a matrix of gene product similarities. Combined with expression data, the matrix is displayed as a set of functional gene networks. The transversal approach was applied to 186 genes related to the enterocyte differentiation stages. This approach resulted in 18 functional networks proved to be biologically relevant. These results were compared with those obtained through a standard approach and with an approach based on information content similarity.

**Conclusion:**

Complementary to the standard approach, the transversal approach offers new insight into the cellular mechanisms and reveals new research hypotheses by combining gene product networks based on semantic similarity, and data expression.

## Background

Interpretation of data resulting from high-throughput analyses is a challenge in bioinformatics. Two major information sources are usually used to make this interpretation: expression data and biological annotations mainly based on the Gene Ontology™ (GO) [1]. According to Eisen et al., expression data organize genes into functional categories [2]. Genes that are expressed together share common functions. Therefore, the interpretation of microarray experimental data is usually performed through the following "standard" approach: 1) the genes are organized into clusters depending on their differential expression pattern and, 2) for each cluster, the main objective is to translate the list of genes into a functional profile able to offer insight into the cellular mechanisms relevant in the given condition [3]. Several tools have been proposed for ontological analysis of gene expression data (for review see [4]). Among them, following the standard approach used to interpret expression data, Gibbons and Roth proposed to judge the quality of the expression-based clustering methods using GO terms [5]. However, as argued in [6,7], complex biological functions emerge from interactions between gene products. Integrated systems, defined as the assembling of individual gene products in such complexes, can collaborate in broader biological processes. For example, in Bacillus subtilis, an ABC transporter and a two-component regulatory system, respectively involved in transport and signal transduction, collaborate into a same biological process: antibiotic resistance [8]. Therefore, if different functions can be involved in a common biological process, we can make the assumption that genes can be differentially expressed in such a process. Consequently, the standard approach makes it difficult to underline functional relationships between gene products when they belong to different expression clusters.

Complementary to the standard approach, we define a transversal analysis that aims to predict functional networks of gene products based on the biological processes they belong to. Simultaneously, genes involved in such networks are clustered according to their expression patterns. The combined visualization of functional networks and expression clusters is expected to offer new insight on the roles of the gene products. We propose to use the ontological-based similarity to predict functional gene product networks. Based on the GO term similarity, the semantic similarity between gene products consists in the comparison of the different terms assigned to a pair of gene products. Typically, two approaches can be performed to compute the term similarity into hierarchies. The path based method relies on the edge-counting approach defined in [9]. The shorter the path one node to the other, the more similar they are. However, the semantic distances between any two adjacent nodes are not necessarily equal. Indeed, the distance shrinks as one descends the hierarchy, since differentiation is based on finer and finer details. The information content method is based on Lin, Jiang and Resnik measures [10-12]. This approach relies on the frequency of a concept in a large corpus. Based on this approach, ongoing works propose to establish functional relationships between gene products [13-16]. As discussed in [17], the information content approach tends to give better results for the term similarities than the path based method. However, applied to the gene similarity, it does not always meaningfully estimate similarity between genes because it does not take into account the hierarchy organizing terms (e.g. [18]).

The transversal approach presented in this paper consists in computing the semantic similarity between gene products in a Vector Space Model [19]. Gene products are described as vectors of GO terms. The major contribution of this approach is the possibility of using a weighting scheme over the annotations. The comparison of such annotation vectors results in a matrix of gene similarity. Combined with expression data, the matrix is displayed as a set of functional gene networks. Each gene-gene relation is associated to the shared annotations. Hierarchy issues are addressed by an 1) *a priori *selection of terms according to a pre-determined level of abstraction and 2) *a posteriori *refinement of data interpretation to focus on a particular biological process. The transversal analysis was applied to a set of differentially expressed genes related to enterocyte differentiation. These genes were previously studied by a standard approach [20].

This paper is organized as follows. First, biological results and their comparison with the KEGG pathways are presented and discussed, then the transversal analysis methodology is detailed.

## Results

### Overview of the transversal approach

**Figure 1:**
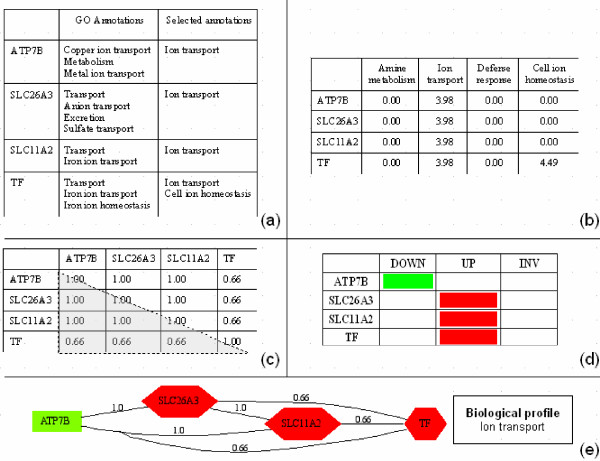
**Transversal analysis steps**. a) The GO terms are selected according to an a priori level of abstraction, b) From these selected terms, term vectors are obtained for each gene products through the application of the idf weighting scheme, c) The comparison of these vectors in a Vector Space Model results in a matrix of similarity, d) Standard expression clustering based methods result in the attribution of each gene products to an expression cluster (up-regulated cluster, down-regulated cluster and invariant cluster i.e. constant expression cluster), e) Based on higher pairwise similarity, the matrix of similarity is displayed as a biological network where the gene expression clustering corresponds to the shape and color of each node. Up-regulated and Down-regulated genes are represented respectively as red hexagons and green boxes.

The standard approach to interpret transcriptomic data aims to retrieve the biological processes mainly involved in a specific condition (for example, mitosis, oncogenesis and proliferation processes are involved in cancer). For this purpose, a collection of differentially expressed genes (up-regulated or down-regulated genes) is characterized by a set of ranked GO terms. Complementary to this approach, the transversal analysis exploits the GO term similarity to cluster the gene products. The behaviors of the resulting networks are analyzed according to the gene expression. Briefly, our method proceeds as follows (see Figure 1):

- starting with a collection of gene products that have been clustered according to their expression with an expression clustering-based method;

- selection of GO terms associated with each gene product according to an *a priori *level of abstraction (*apLev*) (Figure 1a);

- construction of a weighted term vector for each gene product (Figure 1b);

- pairwise comparison of these vectors in a Vector Space Model. This comparison results in a half-matrix of gene product similarities (Figure 1c);

- selection of a similarity threshold to obtain the pairs of gene products that have a high degree of similarity;

- displaying the resulting pairs of gene products associated with their corresponding expression clusters (Figure 1d). A gene product pair is displayed as two nodes linked by an edge. It results in a set of "transversal networks". The most frequent terms are used to describe each network as a biological profile (Figure 1e).

At this step, the resulting networks are biologically interpreted. This analysis can be refined by performing several runs at various levels of abstraction (named *a posteriori *levels). Gene products that are associated with finer-grained GO terms are then grouped together under more general categories.

A detailed description of the methodology is provided in the Methods section.

### Dataset

The transversal analysis was applied to a set of genes related to enterocyte differentiation. These genes were previously studied by a standard approach [20]. In this paper, we refer to this set of genes as the Bedrine-Ferran gene set (BF set). As CaCo-2 cells spontaneously differentiate in enterocytes, this cell line was used to characterize genes whose expression varies during differentiation by means of microarray experiments. The authors performed a clustering with Self-organized Maps (see Methods section) and the resulting expression clusters are used in our approach combined with the transversal networks. These experiments led to the identification of 186 significant genes through the in vitro differentiation process: 50 were down-regulated, 80 up-regulated and 56 were "invariant", *i.e*. their expression remained constant during the differentiation stages. We have applied the transversal analysis to the BF set. 187 distinct Biological Process terms related to 119 gene products were extracted (the 67 remaining gene products were not associated with any GO Biological Process term). As these terms are located at various hierarchy depths, we compared the different levels of abstraction in order to compute, at the most appropriate level, the semantic similarity between the gene products.

### *a priori *level of abstraction

**Figure 2:**
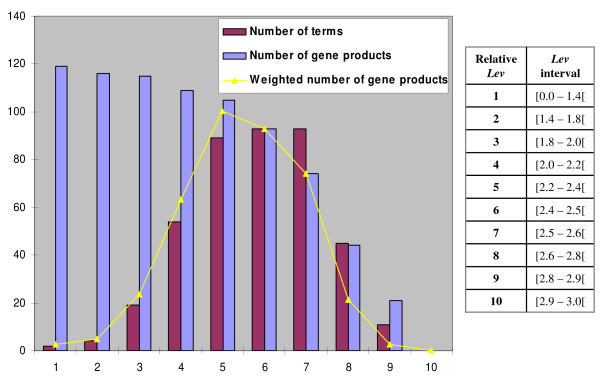
**Number of terms and gene products according to the GO levels**. The table on the right presents the relative level assigning to each *Lev *interval. The yellow curve represents the number of gene products that is weighted by the normalized number of terms. This curve reaches its maximum for the fifth level interval which corresponds then to the *apLev*.

All the ancestors of the 187 terms are retrieved. The augmented set of terms corresponds to 374 terms. For each term, a level (*Lev*) is calculated using the conceptual distance metric approach presented in the Methods section. Terms and consequently gene products are clustered into ten classes, each class corresponding to a *Lev *interval *e.g.*, the first interva1 corresponds to [0–1.4]. As illustrated in Figure 2, the number of annotated gene products decreases for higher values of *Lev*. According to our method, an *a priori *level interval (*apLev*) is computed; it corresponds to the fifth level interval which contains 89 terms associated to 105 gene products. The fifth interval is then selected to compute the gene product similarity.

### Computing semantic similarity

**Figure 3:**
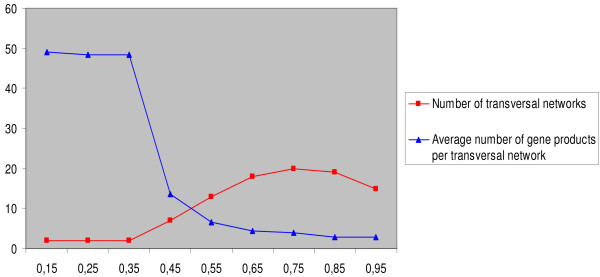
**Selection of a similarity threshold**. Number of networks (red curve) and average number of gene products per network. (blue curve) according to each threshold. The combination of the criteria of selection, i.e. high degree of similarity and high number of gene products per networks, leads us to choose a threshold of .65.

VSM is applied to the BF gene products that are characterized at the fifth GO interval. 105 vectors of GO terms are compared pairwise. From the resulting half-matrix of similarity, we have to select a similarity threshold from which the gene pairs can be displayed in order to obtain the most relevant networks. This selection must take into account the distribution of the networks and the number of gene products per network (see Methods Section). Therefore, we compared the number of networks and the average number of genes per network for different similarity thresholds (see Figure 3). By combining these criteria, we have selected a threshold of .65. We obtain 18 functional networks corresponding to 79 distinct gene products (66% of the BF set). Each network contains 2 to 12 nodes (average of 4.4).

**Figure 4:**
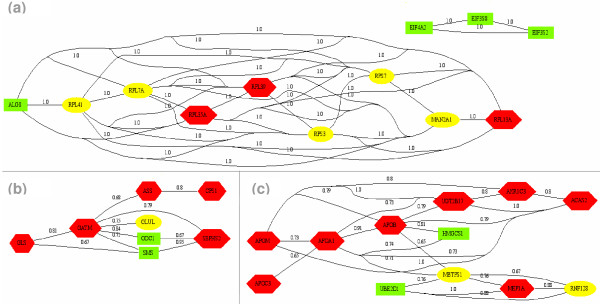
**Results of transversal analysis**. (a) Protein biosynthesis network. While this network is a complete graph, for more clearness, only the highest similarity links are represented. (b) Amine metabolism network. (c) Lipid metabolism and Catabolism. Up-regulated, Down-regulated and invariant genes are represented respectively as red hexagons, green boxes and yellow ellipses.

All the networks are functionally homogeneous, *i.e. *the shared annotations are consistent and the presence of the different gene products in each network has been validated by an expert (See additional file 1: the transversal networks and their biological profiles). Within a given network, some genes are overexpressed, other genes are underexpressed or have an invariant expression (Table 1). All the resulting networks can be visualised on our website [21]. To illustrate our results, we have shown in Figure 4 three networks that have different topologies (e.g. clique or subnetwork definition) and provide novel biological results (such as the finding of a potential new pathway in the enterocyte):

**Table 1:** Biological profiles and gene expression related to the networks of more than two gene products

*Number of genes*	*Biological profile*	*%Down*	*%Up*	*%Inv*
**12**	**cellular macromolecule metabolism; protein metabolism; macromolecule biosynthesis; cellular biosynthesis**	**33**	**25**	**42**
**12**	**cellular lipid metabolism; lipid metabolism; cellular catabolism**	**17**	**66**	**17**
10	organelle organization and biogenesis; DNA metabolism	70	10	20
**8**	**amine metabolism; amino acid and derivative metabolism**	**25**	**62.5**	**12.5**
5	cellular macromolecule metabolism; protein metabolism	60	0	40
4	ion transport	25	75	0
4	cellular catabolism; generation of precursor metabolites and energy; carbohydrate metabolism	50	0	50
3	RNA metabolism	33	33	33
3	intracellular transport; protein transport; establishment of protein localization	75	0	25

Network 1 is depicted in Figure 4a. Its biological profile is protein metabolism/cellular biosynthesis. This network corresponds to a clique and, as described in the Methods section, this topology can be associated to a robust biological network. Three translation initiation factors (EIF4A2, EIF3S2, and EIF3S8), seven ribosomal proteins (RPS7, RPL13A, RPL41, RPL35A, RPL39, RPS3, and RPL7A) and two gene products involved in protein glycosylation (ALG8 and MAN2A1) are involved in this network. With three stages of the protein biosynthesis process – translation initiation, translation and post-translational modification – this network is functionally homogeneous. One might expect, in cellular proliferation, an overexpression of the genes involved in protein biosynthesis. While this is observed for the genes involved in translation initiation, the genes encoding ribosomal proteins are either invariant or down regulated. This down regulation pattern might be related to additional functions (such as transcription, RNA processing, DNA repair, inflammation) as argued in [22,23]. Similarly to translation, initiation of the protein glycosylation in the endoplasmic reticulum might be activated in cell proliferation (down-regulation of ALG8), whereas the later glycosylation steps occurring in the Golgi apparatus might be invariant along the cell differenciation (invariant expression of MAN2A1).

Network 2 is related to the amine metabolism biological profile (Figure 4b). Eight gene products involved in arginine metabolism (GLS, ASS, CPS1 and GLUL), creatine biosynthesis (GATM), polyamine biosynthesis (ODC1 and SMS), and Selenoaminoacid metabolism (SEPHS2) are associated in this network. This network is functionally homogeneous. Its heterogeneous expression profile suggests that a specific biochemical pathway, leading to the creatine precursor (Guanidinoacetate), is activated during differentiation stages (up-regulation of GLS, GATM, ASS and CPS1), while the polyamine biosynthesis is repressed (down-regulation of ODC1 and SMS). For instance, it was proved that only the arginine metabolism is performed in the small intestine [24]. Through this network, a potential NH3-detoxification role could be attributed to the enterocyte. The invariant expression of GLUL is explained by its role – *e.g. *glutamine synthetase – that is opposite to the GLS role – glutaminase – in the amine metabolism. Moreover, the GLUL expression is in accordance with the need of glutamate for complete CaCo-2 cell differentiation [25].

Network 3 is divided into two subnetworks connected by a specific gene product (MBTPS1) involved in both (Figure. 4c). The first subnetwork, lipid metabolism, is functionally homogeneous with nine gene products characterizing the CaCo-2 cells, models for intestinal lipoprotein synthesis and secretion [26]. Four apolipoproteins involved in lipid transport (APOA1, APOB, APOC and APOM) and five gene products involved in cholesterol metabolism (MBTPS1, HMGCS1, ACAS2), androgen metabolism (UGT2B17) and arachidonic acid metabolism (AKR1C3) are gathered through this subnetwork. Seven genes belonging to lipid metabolism appear to be up-regulated, due to the role of diffentiated enterocytes that increase lipid uptake, metabolism and packaging [27]. Conversely, HMGCS1, key enzyme of the cholesterol synthesis, is down-regulated during the differentiation stage. As argued in [28], this enzyme is transcriptionally repressed by an increase of cholesterol in the cell. MBTPS1, through a specific degradation role, is also involved in the cholesterol biosynthesis [29]. The second subnetwork is related to cellular catabolism with three gene products involved in ubiquitine conjugaison (UBE2D1), digestion (MEP1A), and apoptosis (RNF128). MBTPS1, with its degradation role in cholesterol metabolism, is involved in the two subnetworks and represents the only connection between them. All the gene products share a degradation function. Therefore, this subnetwork could be considered as functionally homogeneous according to the catabolism profile. The heterogeneity in gene expression is due to the wide coverage of catabolism. MBTPS1, with its specific role in cholesterol metabolism, represents the only connection between the two subnetworks.

### *a posteriori *level of abstraction

**Figure 5:**
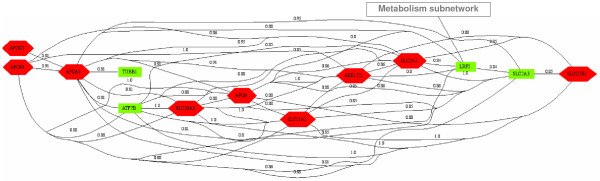
Transport network resulting from an a posteriori level of abstraction run of the transversal analysis.

Among the gene products belonging to the networks obtained in the previous step, some may be involved in a common broader biological process. For example, apolipoproteins, which can be involved in lipid transport, are gathered in the lipid metabolism network (Figure 4c). All would be related to transport along with ion transport gene products (Figure 1e) in a wider network. Therefore, to highlight broader transversal networks, the transversal analysis was applied to GO terms that stand higher in GO hierarchy. The level that is selected is then named *a posteriori *level. The transversal analysis performed with fourth level terms and a threshold of .80 results in 13 biological networks (representing 71 gene products). Among them, a network of 26 gene products consists of two subnetworks of 13 gene products. The first one corresponds to Biosynthesis and the second one to Transport (Figure 5). Only one edge links these subnetworks. Transport genes are mainly up-regulated. According to Bedrine-Ferran et al. [20], when CaCo-2 cells reach confluence and differentiate, they acquire an enterocyte-like phenotype and, as such, they develop the capacity to efficiently transport water, ions, lipids and amino acids.

### KEGG comparison

In order to evaluate the resulting transversal networks, we compared them with the KEGG PATHWAY database which is the reference database as regards the biochemical pathways (including most of the known metabolic pathways and some of the regulatory pathways)[30]. KEGG pathways are structured according to a three-level hierarchy including six major root classes: *Metabolism*, *Genetic Information Processing*, *Environmental Information Processing*, *Cellular Processes*, *Human Diseases and Drug Development*. The third level of this hierarchy corresponds to the KEGG pathways. A relation between a third level term and a gene product in the KEGG pathway database is considered as a KEGG annotation. The second level terms correspond to broader biological pathways.

A KEGG local relational database was built from the hierarchy available from the KEGG website. The database was used for querying and evaluating the transversal networks. The comparison was manually done.

Each transversal network was compared with the KEGG pathways. Among the 79 gene products resulting from the transversal analysis, 43 are annotated with KEGG. These gene products are associated with 49 pathways. Among the 18 transversal networks, 10 can be evaluated, *i.e. *they are composed of at least two gene products present in KEGG. Six transversal networks were consistent with KEGG: in four cases, KEGG annotations are identical or correspond to sibling KEGG pathways (*i.e. *pathways are subsumed by the same two-level term); for one network, KEGG annotations correspond to closely related two-level terms (Amino acid metabolism/Metabolism of others amino acids); in one case, KEGG annotations are different but reflect the composition of the networks into subnetworks (in network 3, gene products are annotated with *Lipid metabolism *and *Folding*, *Sorting and Degradation*).

The four remaining networks are heterogenous. However, from a biological point of view, the KEGG annotations are complementary. For example, the network 1 is associated with *Translation*, *Transcription *and *Glycan Biosynthesis and metabolism*. While the Translation and the Transcription derive from *Genetic Information Processing*, the last one corresponds to *Metabolism *although this pathway is related to the post-translation modifications.

An extract of the KEGG comparison is given in the Table 2. All the comparison results are given in the supplementary file 2: KEGG comparison.

**Table 2:** KEGG results for the three networks

*Transversal analysis network*	*Gene products*	*KEGG classification*
**Network 1** Protein metabolism; Cellular biosynthesis	RPL13A-RPL7A-RPL35A-RPL39-RPL41-RPS3-RPS7 EIF4A2-EIF3S2-EIF3S8 ALG8-MAN2A1	Translation Transcription Glycan Biosynthesis and metabolism
**Network 2** Amine metabolism	GLS-GLUL-CPS1-GATM-ASS-ODC1-SMS-SEPHS2	Amino Acid Metabolism Metabolism of Other Amino Acids
**Network 3** Lipid metabolism; Cellular catabolism	HMGCS1-AKR1C3-UGT2B1-ACAS2 UBE2D1	Lipid Metabolism Folding, Sorting and Degradation

## Discussion

This paper presents a new approach to microarray data interpretation which combines gene product interaction networks with data expression and offers new insight into the cellular mechanisms. The biological networks obtained by this approach rely on ontology-based similarity which is computed through the VSM. We first presented a preliminary study of this approach in [31] and we have enriched it with the definition of a conceptual distance metric. This improvement of the path approach is used to select the GO Biological Process terms in each annotation vector. The transversal analysis was applied to a collection of genes related to the enterocyte differentiation. 18 functional networks involving 79 gene products were obtained. The 26 unrepresented gene products present a similarity degree that is under the selected threshold. To measure the significance of the gene product networks resulting from the transversal analysis, recall and precision were calculated by comparing the gene product classification resulting from our method against a gold standard: the KEGG pathway database. We estimated the precision to be 81.8% and the recall to be 83.7%. Moreover, the "false positives" were judged biological relevant by the experts, proving that our method provides the biologists with valuable information. The "false negatives" were due to the incompleteness of the Gene Ontology annotation. Furthermore, the experts considered the resulting networks to be functionally homogeneous. The gene expression differs within each network (Table 1), highlighting that some specific processes are activated under some conditions. For example, network 2 suggests a new potential pathway related to amine metabolism that could be activated during the differentiation stage. This result emphasizes the contribution of the transversal analysis to suggest new research hypotheses. Furthermore, while the transversal network 1 (protein biosynthesis) presents an expression heterogeneity which is biologically relevant, the standard approach did not highlight this expression fluctuation during the cellular differentiation process (See additional file 3: Standard approach comparison). The graphviz software is used to visualize the resulting networks [32]. Coupling this software with web technology assists the biological interpretation of transversal networks by associating each node (gene product) to its GO annotations and to the GO terms that are shared by all the gene products (networks are presented on a web page where the annotations are reachable by clicking on each node). By using different values for GO interval and threshold, the visualization of the networks is kept readable even if the number of studied gene products increases. While our method aims to retrieve all the networks related to dedicated microarrays, different strategies can be achieved in the case of pangenomic microarrays. For example, the method can be used to retrieve specific networks associated to fine-grained terms (by increasing the *Lev *parameter). Moreover, a first run can select the networks of interest which can be refined by a second run.

The networks obtained by our method must contain enough gene products to cover biological pathways. One criterion used to select the similarity threshold is the number of gene products per network. However, even two-gene networks may be biologically relevant. For example, the opposite expression of the two genes involved in the network 15 (SLC2A3-down and SLC2A5-up in additional file 1) is discussed in [33]. Other approaches to compute gene product similarity based on GO have been developed, e.g. the Bioconductor system proposes an index which is similar to the Jaccard similarity coefficient used for comparing the similarity of sample sets [34,35]. Although these approaches are useful to obtain a preliminary analysis of a set of proteins (as it is the case in [35]), they lack in some widespread conditions, such as intensive annotation of a gene product, intensive use of a term to annotate a set of gene products or difference in the term granularity associated with the gene products to be compared. Among the more complex approaches using GO to compute gene product similarity, Azuaje et al. proposed a method based on the maximum inter-set similarity between terms to address the hierarchy limitation [18]. We have compared our networks to those obtained by the Azuaje method applied to Lin's similarity with a threshold of .65 (selected by combining the same criteria as for our analysis. See the Results section for the criteria and the additional file 4 for the selection). Rationale for choosing Lin's similarity was that the results vary from 0 to 1. The comparison between the Lin's networks and the transversal networks has shown that the transversal analysis gives better results. Indeed, among the resulting 12 Lin's networks, two are identical to those found through the transversal analysis and seven combine several distinct networks, resulting in heterogeneous networks (Table 3, network 1) and/or incomplete ones (Table 3, network 2). While distance based on information content depends on literature, VSM weighting decreases the importance of processes that are over represented in the set. Therefore, the transversal analysis appears more discriminant (See additional file 5: Azuaje comparison).

**Table 3:** Major results with Azuaje method. Azuaje approach results in 52 gene products categorized as 12 networks from two to 14 gene products. The first Azuaje network merges two transversal networks. The gene products belonging to the transversal network 3 are emphasized with bold-face.

***Azuaje network***	***Gene products***	***Transversal results***	***GO profiles***
1	RPL7A-RPL39-RPL41-RPS3-RPS7-EIF4A2-EIF3S2-EIF3S8-ALG8-MAN2A1-**HMGCS1-UGT2B17-AKR1C3-ACAS2**	Network 1 + **Network 3**	Protein metabolism; Cellular Biosynthesis; Lipid metabolism
2	GATM-ODC1-SMS-SEPHS2	Partial Network 2 (50%genes products missing)	Amine metabolism

To support the prediction role of the transversal approach, we take into account all the GO terms that are associated with gene products in annotation databases whatever the evidence codes. Indeed, taking into account only non-IEA (not inferred from electronic annotation) codes could result in using only 40% of the annotations provided by the Gene Ontology Annotation database (GOA; [36]). Therefore, we have chosen to favor a high number of biological assumptions rather than the reliability of annotations. In the same way, the GO interval addresses the issue of the various GO branch depth by selecting annotations independently of the tree depth.

The *a posteriori *level of abstraction section shows the importance of selecting the granularity to study biological processes. To our knowledge, this granularity is not taken into account in other approaches based on the GO semantic similarity. The way to restrict GO terms in our approach is complementary to using a GO slim [37] to perform analysis. Indeed, while GO slims are mainly domain-dependent, the restriction that is performed in our approach depends on the set of terms that annotate the gene products and their level in the GO hierarchy.

The GO annotation is remarkably useful for the mining of functional and biological significance from large datasets, such as microarray results [38]. However, the transversal analysis results reflect some gaps in GO annotation databases. Indeed, the network 1 (protein metabolism/cellular biosynthesis) includes some ribosomal proteins. While some publications confirm that these gene products can be involved in replication, DNA repair or inflammation processes, there is no relation between these processes and the ribosomal proteins in GOA. The type of process itself can also cause difficulty in network interpretation. Some transversal high-level processes (*e.g. *catabolism in the network 3), gather gene products that are involved in several processes (*e.g. *apoptosis or digestion for this network).

The KEGG hierarchy is used to classify the biochemical pathways. However, the lack of relation between the KEGG root classes can introduce a bias. Indeed, translation and post-translation modification correspond to independent classes in the KEGG hierarchy (Genetic Information Processing and Metabolism). With regards to these results, we consider using the GO relations found in the resulting networks to enrich our KEGG local database. While 79 gene products are represented in the 18 transversal analysis networks, 43 are present in 49 KEGG pathways. Whereas the relative high number of pathways is partly due to a finer granularity of their description, some of them are not present in the GO (such as the Human Diseases class related pathways). Therefore, we consider taking into account the KEGG data in the transversal approach. In addition, we will have to evaluate the relative contribution of GO and KEGG vocabularies in order to weight the terms during the VSM step of the transversal analysis. By adding KEGG terms, we expect to improve the definition of the transversal networks.

Currently, we are working on an improvement of the transversal approach by weighting the levels according to the local density of each node as suggested in [39]. Future work will consist in comparing and possibly merging our approach with literature networks as described in [40]. Furthermore, we plan to consider a measure of functional diversity, as the functional entropy discussed in [41], in order to support the evaluation of the transversal networks.

## Conclusion

This paper presents a new approach to microarray result interpretation which aims to combine gene product interaction networks with data expression. The resulting transversal networks are proved to be biologically consistent and offer new insight into the cellular mechanisms. Furthermore, the comparison with a standard approach corroborates the contribution of the transversal approach and underlines the complementarity of these two approaches.

Two major points reflect the novelty of the methodology developed to construct the transversal networks: the selection of an annotation level and the use of a weighting scheme over the annotations prior to compute the semantic similarity. The former point avoids the artefact due to the arbitrary fluctuation of the GO depth and in addition takes into account the granularity of the studied biological processes. The latter point considers the representativity of the annotations associated with the set of studied gene products. The comparison with gene products clustering derived from an approach based on information content highlights the contribution of the transversal approach to the construction of gene product networks. Finally, the comparison with the use of a biological vocabulary differently structured (*i.e. *KEGG hierarchy) proved that using a weighting scheme over the GO annotations and computing the similarity between gene products in a VSM constitute an efficient mean to construct gene product networks and consequently to interpret microarray results.

## Methods

The GO terms are organized according to three independent hierarchies: Biological Process, Molecular Function, and Cellular Component. The transversal analysis uses the Gene Ontology Annotation file (GOA; [36]) to provide assignments of GO terms to gene products. It is restricted to terms from the Biological Process hierarchy in order to retrieve genes functionally related to a given biological process.

### Computing gene similarities

A Vector Space Model (VSM) is used to compute similarity between pairs of gene products. VSM are essentially used in information retrieval for computing the similarity between documents described as vectors of keywords. Recently, this method has been used to identify associative relations between terms in the GO [42]. The transversal analysis uses VSM to compute the similarity between gene products described as vectors of GO terms. A gene product is represented by a specific vector g as follows:

*g *= (*t*_1_, *t*_2_,..., *t_n_*)

Where *t*_*i *_is the numeric value that the term *i *takes on for this gene product and *n *is the number of GO terms associated with the set of gene products. For example, *t*_*i *_= 0 when there is no association between the GO term and the gene product in GOA. Since different terms have different importance for a gene product, a term weight is associated with each term. Lower weights are assigned to less important terms. In standard VSM, a common approach uses the *idf *method in which the weight of a term is determined by the way this term occurs in the whole document collection (inverse document frequency) [43]. In the case of a gene product collection, we consider that a term is not representative of a gene product if it annotates most of the gene products in the collection. The term weight (*w*_*t*_) is inversely proportional to the ratio of the number of gene products annotated by the term *t *(*n*_*t*_) to the total number of annotated gene products in the collection (*N*):

*w_t_*= *idf_t_*= log*N*/*n_t_*

Once the term weights are determined, a gene product is represented by the following specific vector:

*g *= (*w*_1_, *w*_2_,..., *w_n_*)

Figure 1b shows an example of weighted annotation vectors. A vector inner-product function can be used to compute term overlap between any two gene product vectors. When gene products are associated with numerous different GO terms, the similarity between these gene products is higher than expected, compared to the similarity between gene products that are annotated by fewer GO terms. To compensate for this effect, normalization of term weights is used. Normalization is a way of imposing some penalty on the term weight for high annotated gene products. Cosine normalization is an effective normalization technique. It corresponds to the division of each gene product vector by its Euclidean length [44]. Let *n *be the number of terms that annotate the genes in the collection, given a pair of gene products, *g*1 and *g*2, the semantic similarity, *Sim(g1, g2)*, is defined as follows:

Sim(g1,g2)=g1→.g2→|g1||g2|=∑i=1nw1i×w2i∑i=1n(w1i)²×(w2i)²
 MathType@MTEF@5@5@+=feaafiart1ev1aaatCvAUfKttLearuWrP9MDH5MBPbIqV92AaeXatLxBI9gBaebbnrfifHhDYfgasaacH8akY=wiFfYdH8Gipec8Eeeu0xXdbba9frFj0=OqFfea0dXdd9vqai=hGuQ8kuc9pgc9s8qqaq=dirpe0xb9q8qiLsFr0=vr0=vr0dc8meaabaqaciaacaGaaeqabaqabeGadaaakeaacqWGtbWucqWGPbqAcqWGTbqBcqGGOaakcqWGNbWzliabigdaXOGaeiilaWIaem4zaC2ccqaIYaGmkiabcMcaPiabg2da9maalaaabaWaa8HaaeaacqWGNbWzliabigdaXaGccaGLxdcacqGGUaGldaWhcaqaaiabdEgaNTGaeGOmaidakiaawEniaaqaamaaemaabaGaem4zaC2ccqaIXaqmaOGaay5bSlaawIa7amaaemaabaGaem4zaC2ccqaIYaGmaOGaay5bSlaawIa7aaaacqGH9aqpdaWcaaqaamaaqahabaGaem4DaC3ccqaIXaqmdaWgaaadbaGaemyAaKgabeaakiabgEna0kabdEha3TGaeGOmaiZaaSbaaWqaaiabdMgaPbqabaaaleaacqWGPbqAcqGH9aqpcqaIXaqmaeaacqWGUbGBa0GaeyyeIuoaaOqaamaakaaabaWaaabCaeaacqGGOaakcqWG3bWDliabigdaXmaaBaaameaacqWGPbqAaeqaaOGaeiykaKIaeiOSaiRaey41aqRaeiikaGIaem4DaC3ccqaIYaGmdaWgaaadbaGaemyAaKgabeaakiabcMcaPiabcklaYcWcbaGaemyAaKMaeyypa0JaeGymaedabaGaemOBa4ganiabggHiLdaakeqaaaaaaaa@779D@

The semantic similarity between two gene products varies from 0 (no similarity) to 1 (complete similarity). Similarity is computed pairwise for all the gene products of the collection. It results in a half-matrix of gene product similarity. A threshold must be chosen for the inner product in order to select the pairs of gene products that present a high degree of similarity. This threshold has to 1) select the pairs of genes that have a high degree of similarity, 2) result in a high number of networks and 3) have biological significance: each network must contain enough gene products to offer new insight into cellular mechanisms. Each gene product involved in the half-matrix is associated with the expression cluster it belongs to. These expression clusters can result from : 1) an expression clustering-based method that can be combined with our transversal approach (e.g. hierarchical clustering, k-means clustering or Self-Organized Maps (SOM) which are the most widely used in analysis of gene-gene expression data (for review see [45]), 2) a specific database (as Gene Expression Omnibus; [46]), or 3) the literature.

The gene product pairs that show a degree of similarity higher than the threshold are displayed as two nodes linked by an edge. This results in a set of functional networks. We use Graphviz [32], an open source software developed at AT&T Labs, in order to visualize these networks. The node shape and color correspond to the expression cluster each gene belongs to. An edge between two nodes is associated with the degree of similarity between two gene products according the given threshold. Each edge is typed by the GO terms shared by the two gene products. A biological profile is defined for each network; it corresponds to the most frequent terms typing the edges of a network. The network interpretation is firstly based on graph theory, for example a network is named a clique (or complete graph) if each pair of nodes is joined by an edge. Applied to gene products, this graph property highlights a robust biological network. On the other hand, the biological profiles assist the evaluation of the resulting networks.

### Conceptual distance metric

VSM considers GO terms as independent. Therefore, when two gene products are associated with terms that are parent and child in the GO hierarchy (e.g. ATP7B and SLC26A3 in Figure 1a), the hierarchical relation is not taken into account. Moreover, the depth of the GO hierarchy varies from 2 nodes to 15 nodes depending on the branches. Some of the variation is inherent in different functional families, while some may be an artefact of the uneven contribution by different groups participating in GO's development [47]. Therefore, considering all annotations assigned to a gene product within a hierarchy can introduce a bias when two gene products are associated to different-level annotations. In order to address this issue, we have taken into account multiple-level GO annotations, which derive from a recursive definition of ancestors, all the way to the top of the hierarchy. The ancestors of each GO term associated with a gene product are retrieved. An annotation level is then selected to compute semantic similarity. However, as a GO term may have more than one parent, the level of a term may be different depending on the path toward the top of the hierarchy. To assign a fixed level to each GO term, we use a metrics based on the edge-counting approach defined in [9]. In this approach, conceptual distance is calculated by counting the number of nodes, each node representing a distinct term. As proposed in [11], terms in a deeper part of the hierarchy should be ranked closer. Based on this assumption, we use the formula described in [48] to assign a level value to each term. Let *depth*(*t*) be the edge distance from the root term to the term *t*. r is the number of paths from the root term to a term *t *and m_j _the number of terms in a path j. The level of a term *t*, *Lev*(*t*), is defined as follows:

Lev(t)=min⁡j∈{1..r}∑i=1mj1/depth(tj.i)
 MathType@MTEF@5@5@+=feaafiart1ev1aaatCvAUfKttLearuWrP9MDH5MBPbIqV92AaeXatLxBI9gBaebbnrfifHhDYfgasaacH8akY=wiFfYdH8Gipec8Eeeu0xXdbba9frFj0=OqFfea0dXdd9vqai=hGuQ8kuc9pgc9s8qqaq=dirpe0xb9q8qiLsFr0=vr0=vr0dc8meaabaqaciaacaGaaeqabaqabeGadaaakeaacqWGmbatcqWGLbqzcqWG2bGDcqGGOaakcqWG0baDcqGGPaqkcqGH9aqpdaGfqbqabSqaaiabdQgaQjabgIGiopaaceaabaGaeGymaeJaeiOla4IaeiOla4YaaiGaaeaacqWGYbGCaiaaw2haaaGaay5EaaaabeGcbaGagiyBa0MaeiyAaKMaeiOBa4gaamaaqahabaWaaSGbaeaacqaIXaqmaeaacqWGKbazcqWGLbqzcqWGWbaCcqWG0baDcqWGObaAcqGGOaakcqWG0baDliabdQgaQjabc6caUiabdMgaPPGaeiykaKcaaaWcbaGaemyAaKMaeyypa0JaeGymaedabaGaemyBa02aaSbaaWqaaiabdQgaQbqabaaakiabggHiLdaaaa@59CF@

*Lev *values are calculated for all the GO terms associated with the collection of gene products. The space of *Lev *values is divided into ten intervals. Each interval must contain only one value of *Lev*(*t*) in order to avoid the integration of hierarchical related terms in the vectors used to compute semantic similarity. The most informative level interval has to be selected to compute the semantic similarity between the genes. While deepest levels in the hierarchy contribute to a better characterization of gene products, each specific category does not appear to be significant because there are only few gene products associated with it.

Therefore, we introduce the *a priori *level interval (*apLev*) which corresponds to the best compromise between the number of annotated gene products and the number of terms per level interval. *apLev *is computed during a first run of the transversal analysis. For a level interval *j*, let *Tj *and *Gj *be respectively, the number of terms and the number of gene products. We use the normalized *Tj *as a weighting coefficient for the *Gj*. The *a priori *level interval is the one for which the weighted *Gj *is maximum:

apLev=i∈{1;..;10}|∀j∈{1;..;10} αjTjGj≤αiTiGi     with∀j∈{1..10} αj=1/max⁡k=110(Tk)
 MathType@MTEF@5@5@+=feaafiart1ev1aaatCvAUfKttLearuWrP9MDH5MBPbIqV92AaeXatLxBI9gBaebbnrfifHhDYfgasaacH8akY=wiFfYdH8Gipec8Eeeu0xXdbba9frFj0=OqFfea0dXdd9vqai=hGuQ8kuc9pgc9s8qqaq=dirpe0xb9q8qiLsFr0=vr0=vr0dc8meaabaqaciaacaGaaeqabaqabeGadaaakeaafaqaaeGabaaabaWaaqGaaeaacqWGHbqycqWGWbaCcqWGmbatcqWGLbqzcqWG2bGDcqGH9aqpcqWGPbqAcqGHiiIZcqGG7bWEcqaIXaqmcqGG7aWocqGGUaGlcqGGUaGlcqGG7aWocqaIXaqmcqaIWaamcqGG9bqFaiaawIa7aiabgcGiIiabdQgaQjabgIGiolabcUha7jabigdaXiabcUda7iabc6caUiabc6caUiabcUda7iabigdaXiabicdaWiabc2ha9jabbccaGGGaciab=f7aHjabdQgaQjabdsfaujabdQgaQjabdEeahjabdQgaQjabgsMiJkab=f7aHjabdMgaPjabdsfaujabdMgaPjabdEeahjabdMgaPbqaaiaaxMaacaWLjaGaaCzcaiaaxMaafaqabeqacaaabiabba9=bGbabGmecaoecqqG3bWDcqqGPbqAcqqG0baDcqqGObaAaeaadaWcgaqaaiabgcGiIiabdQgaQjabgIGiolabcUha7jabigdaXiabc6caUiabc6caUiabigdaXiabicdaWiabc2ha9jabbccaGiab=f7aHjabdQgaQjabg2da9iabigdaXaqaamaaxadabaGagiyBa0MaeiyyaeMaeiiEaGhaleaacqWGRbWAcqGH9aqpcqaIXaqmaeaacqaIXaqmcqaIWaamaaGccqGGOaakcqWGubavcqWGRbWAcqGGPaqkaaaaaaaaaaa@8D0A@

The transversal analysis can be refined by performing several runs repeatedly, at various *a posteriori *levels of abstraction. Gene products that are associated with finer-grained GO terms are then grouped together under more general categories. In addition to retrieving more significant terms, such *a posteriori *levels can be used to focus on broader biological processes (e.g. ion transport rather than cation transport, anion transport, etc.).

## Authors' contributions

JC designed and developed the transversal approach. JM evaluated the biological results. AB supervised this study. All authors read and approved the final manuscript.

## Supplementary Material

Additional file 1 **The transversal networks and their biological profiles**. This file contains a table presenting the gene products involved in each network and the corresponding GO profiles. These profiles are ranked according to the number of occurrences of the shared terms. Click here for file

Additional file 2 **KEGG comparison**. This file contains a table presenting the KEGG annotations (level 2 of the KEGG hierarchy) associated with each transversal network and their GO profiles. Click here for file

Additional file 3 **Standard approach comparison**. This file contains a comparison of the transversal approach and the standard approach described in Bedrine-ferran's work. Click here for file

Additional file 4 **Selection of a similarity threshold for the Azuaje's approach**. This file contains a graph representing the number of networks (red curve) and average number of gene products per network. (blue curve) according to each threshold. The combination of the criteria of selection, i.e. high degree of similarity and high number of gene products per networks, leads us to choose a threshold of .65 for the Azuaje's approach. Click here for file

Additional file 5 **Azuaje comparison**. This file contains a table presenting the networks obtained from the Azuaje methodology associated with their corresponding transversal networks (or part of networks) and their GO profiles. Click here for file
