# Supplementary material for: A transversal approach to predict gene product networks from ontology-based similarity
Source: BMC Bioinformatics. 2007 Jul 2;8:235. doi: 10.1186/1471-2105-8-235 (PMC1940024; doi:10.1186/1471-2105-8-235)
Supplement: Additional file 1 — The transversal networks and their biological profiles. This file contains a table presenting the gene products involved in each network and the corresponding GO profiles. These profiles are ranked according to the number of occurrences of the shared terms. [file 1471-2105-8-235-S1.pdf]

A transversal approach to predict gene product networks from ontology-based similarity

Julie Chabalier, Jean Mosser and Anita Burgun

Supplementary information: Main results – GO profiles

Network	Gene product	Gene Ontology profile<br>(occurrence number of shared terms)
**1**	ALG8 - RPL41 - RPL7A -RPL35A - RPL39 - RPS3 -RPS7 - MAN2A1 - RPL13A - EIF4A2 - EIF3S8 - EIF3S2	cellular biosynthesis(66)<br>cellular macromolecule metabolism(66)<br>macromolecule biosynthesis(66)<br>protein metabolism(66)<br>regulation of biosynthesis(3)
**2**	GLS - GATM - ASS -CPS1 – GLUL - ODC1 - SEPHS2 - SMS	amine metabolism(10)<br>amino acid and derivative metabolism(10)<br>organic acid metabolism(8)<br>cellular biosynthesis(6)<br>nitrogen compound biosynthesis(1)<br>sulfur metabolism(1)<br>arginine metabolism(1)
**3**	APOM - APOC3 - APOA1 -APOB<br>UGT2B17 - AKR1C3 - ACAS2<br>HMGCS1 - MBTPS1 - UBE2D1<br>MEP1A - RNF128	lipid metabolism(18)<br>cellular lipid metabolism(17)<br>cellular macromolecule metabolism(8)<br>protein metabolism(8)<br>biopolymer catabolism(6)<br>cellular catabolism(6)<br>macromolecule catabolism(6)<br>alcohol metabolism(5)
**4**	HMGA2 -H3F3B - H2AFY - NAP1L4<br>NAP1L1 - MCM3 - TRIM28 - PIAS4<br>G22P1 - HMGB1	nucleobase, nucleoside, nucleotide and nucleic acid metabolism(14)<br>DNA metabolism(12)<br>organelle organization and biogenesis(10)<br>cellular macromolecule metabolism(7)<br>protein metabolism(7)<br>regulation of nucleobase, nucleoside, nucleotide and nucleic acid metabolism(5)<br>response to DNA damage stimulus(1)
**5**	CS - GAPD - LDHB -ACO1	generation of precursor metabolites and energy(4)<br>carbohydrate metabolism(4)<br>cellular catabolism(4)<br>cellular macromolecule metabolism(4)<br>cofactor metabolism(1)<br>alcohol metabolism (1)<br>macromolecule catabolism(1)
**6**	ATP7B - SLC26A3 - SLC11A2 - TF	ion transport(6)
**7**	VAPB - HSPA9B - TRIP12 - NKTR<br>TRAP1	cellular macromolecule metabolism(10)<br>protein metabolism (10)
**8**	HSPD1 - KPNB1 - CALR	protein transport(3)<br>establishment of protein localization(3)<br>intracellular transport(3)<br>cellular macromolecule metabolism(1)<br>protein metabolism(1)

**9**	RNASE4 -IVNS1ABP - SF3B2 RNA	metabolism (3)<br>nucleobase, nucleoside, nucleotide and nucleic acid metabolism (3)
**10**	KRT8 -TUBA1	organelle organization and biogenesis
**11**	NME2 - NME1	nucleobase, nucleoside, nucleotide and nucleic acid metabolism<br>cellular biosynthesis<br>negative regulation of cellular physiological process<br>regulation of cell proliferation<br>regulation of cell cycle
**12**	ADH4 - GPD1	alcohol metabolism
**13**	FN1 - SERPINA1	defense response<br>response to pest, pathogen or parasite<br>response to external biotic stimulus
**14**	ZW10 - MMS19L	M phase<br>mitotic cell cycle<br>sister chromatid segregation
**15**	SLC2A5 - SLC2A3	carbohydrate transport
**16**	CDC2 - STK6	phosphorus metabolism<br>cellular macromolecule metabolism<br>protein metabolism<br>M phase<br>mitotic cell cycle
**17**	AKR1C1 - NDRG1	response to abiotic stimulus
**18**	VTN - AFP	defense response
