# Supplementary material for: A transversal approach to predict gene product networks from ontology-based similarity
Source: BMC Bioinformatics. 2007 Jul 2;8:235. doi: 10.1186/1471-2105-8-235 (PMC1940024; doi:10.1186/1471-2105-8-235)
Supplement: Additional file 2 — KEGG comparison. This file contains a table presenting the KEGG annotations (level 2 of the KEGG hierarchy) associated with each transversal network and their GO profiles. [file 1471-2105-8-235-S2.pdf]

A transversal approach to predict gene product networks from ontology-based similarity

Julie Chabalier, Jean Mosser and Anita Burgun

Supplementary information: KEGG comparison

Network	Gene product	Gene Ontology profile	Kegg gene product	Kegg annotation<br>*(Level 2)*
1	ALG8 - RPL41 - RPL7A - RPL35A - RPL39 - RPS3 - RPS7 - MAN2A1 - RPL13A - EIF4A2 - EIF3S8 - EIF3S2	cellular biosynthesis(66)<br>cellular macromolecule metabolism(66)<br>macromolecule biosynthesis(66)<br>protein metabolism(66)<br>regulation of biosynthesis(3)	RPL41 - RPL7A - RPL35A - RPL39 - RPS3 - RPS7 - RPL13A	Translation
			EIF4A2- EIF3S8- EIF3S2	Transcription
			ALG8 - MAN2A	Glycan Biosynthesis and Metabolism
2	GLS - GATM - ASS - CPS1 - GLUL - ODC1 - SEPHS2 - SMS	amine metabolism(10)<br>amino acid and derivative metabolism(10)<br>organic acid metabolism(8)<br>cellular biosynthesis(6)<br>nitrogen compound biosynthesis(1)<br>sulfur metabolism(1)	GLS - GATM – ASS - CPS1 – GLUL - ODC1 - SEPHS2 - SMS	Amino acid metabolism<br>Metabolism of others amino acids

		arginine metabolism(1)		
3	APOM - APOC3 - APOA1 - APOB - UGT2B17 - AKR1C3 - ACAS2 - HMGCS1 - MBTPS1 - UBE2D1 - MEP1A - RNF128	lipid metabolism(18)<br>cellular lipid metabolism(17)<br>cellular macromolecule metabolism(8)<br>protein metabolism(8)<br>biopolymer catabolism(6)<br>cellular catabolism(6)<br>macromolecule catabolism(6)<br>alcohol metabolism(5)	UGT2B17 - AKR1C3 - HMGCS1 – ACAS2	Lipid metabolism
			UBE2D1	Folding, Sorting and Degradation
4	HMGA2 - H3F3B - H2AFY - NAP1L4 - NAP1L1 - MCM3 - TRIM28 - PIAS4 - G22P1 - HMGB1	nucleobase, nucleoside, nucleotide and nucleic acid metabolism(14)<br>DNA metabolism(12)<br>organelle organization and biogenesis(10)<br>cellular macromolecule metabolism(7)<br>protein metabolism(7)<br>regulation of nucleobase, nucleoside, nucleotide and nucleic acid metabolism(5)<br>response to DNA damage stimulus(1)	MCM3	Cell Growth and Death
			PIAS4	Signal Transduction
5	CS - GAPD - LDHB - ACO1	generation of precursor metabolites and energy(4)<br>carbohydrate metabolism(4)<br>cellular catabolism(4)<br>cellular macromolecule metabolism(4)<br>cofactor metabolism(1)<br>alcohol metabolism (1)	CS - LDHB - ACO1	Carbohydrate Metabolism

		macromolecule catabolism(1)		
6	ATP7B - SLC26A3 - SLC11A2 - TF	ion transport(6)	none	none
7	VAPB - HSPA9B - TRIP12 - NKTR - TRAP1	cellular macromolecule metabolism(10)<br>protein metabolism (10)	HSPA9B	Signal Transduction<br>Immune System
8	HSPD1 - KPNB1 - CALR	protein transport(3)<br>establishment of protein localization(3)<br>intracellular transport(3)<br>cellular macromolecule metabolism(1)<br>protein metabolism(1)	HSPD1	Metabolic Disorders<br>Neurodegenerative Disorders
			CALR	Immune System
9	RNASE4 - IVNS1ABP - SF3B2	RNA metabolism (3)<br>nucleobase, nucleoside, nucleotide and nucleic acid metabolism (3)	none	none
10	KRT8 -TUBA1	organelle organization and biogenesis	KRT8 - TUBA1	Cell Communication
11	NME2 - NME1	nucleobase, nucleoside, nucleotide and nucleic acid metabolism<br>cellular biosynthesis<br>negative regulation of cellular physiological process<br>regulation of cell proliferation<br>regulation of cell cycle	NME1 - NME2	Nucleotide Metabolism
12	ADH4 - GPD1	alcohol metabolism	ADH4 - GPD1	Lipid metabolism
13	FN1 - SERPINA1	defense response<br>response to pest, pathogen or	FN1	Cell Communication<br>Cell Motility

		parasite<br>response to external biotic stimulus		Signaling Molecules and Interaction
			SERPINA1	Immune System
14	ZW10 - MMS19L	M phase<br>mitotic cell cycle<br>sister chromatid segregation	none	none
15	SLC2A5 - SLC2A3	carbohydrate transport	none	none
16	CDC2 - STK6	phosphorus metabolism<br>cellular macromolecule metabolism<br>protein metabolism<br>M phase<br>mitotic cell cycle	CDC2	Cell Growth and Death<br>Cell Communication
17	AKR1C1 - NDRG1	response to abiotic stimulus	AKR1C1	Xenobiotics Biodegradation and Metabolism
18	VTN - AFP	defense response	VTN	Cell Communication<br>Signaling Molecules and Interaction
