# Supplementary material for: A transversal approach to predict gene product networks from ontology-based similarity
Source: BMC Bioinformatics. 2007 Jul 2;8:235. doi: 10.1186/1471-2105-8-235 (PMC1940024; doi:10.1186/1471-2105-8-235)
Supplement: Additional file 3 — Standard approach comparison. This file contains a comparison of the transversal approach and the standard approach described in Bedrine-ferran's work. [file 1471-2105-8-235-S3.pdf]

A transversal approach to predict gene product networks from ontology-based similarity

Julie Chabalier, Jean Mosser and Anita Burgun

Supplementary information: Standard approach comparison

Bedrine-Ferran H, Le Meur N, Gicquel I, Le Cunff M, Soriano N, Guisle I, Mottier S, Monnier A, Teusan R, Fergelot P *et al*: **Transcriptome variations in human CaCo-2 cells: a model for enterocyte differentiation and its link to iron absorption**. *Genomics* 2004, **83**(5):772-789.

Fig. 4. Process keywords remaining after inspection.

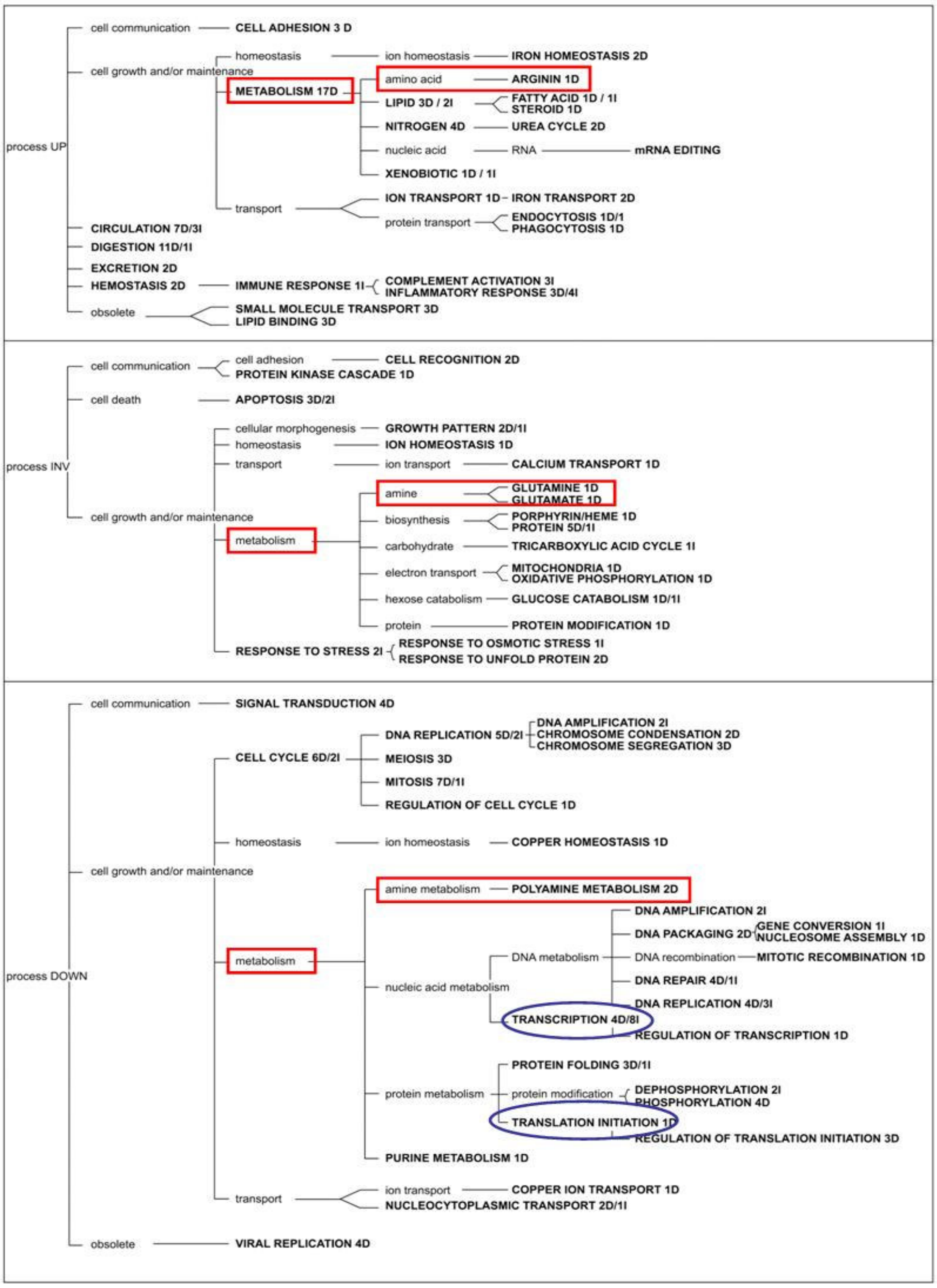

Comparison with the transversal approach:

- While the transversal analysis suggests new research trends through the network 2 related to the Amine metabolism, Bedrine-Ferran et al. report that the amine metabolism process is involved in the three expression clusters during the differentiation stage (See red rectangles on the figure).

- While the transversal network 1 (protein biosynthesis) presents an expression heterogeneity biologically relevant, the two-step approach did not highlighted this expression fluctuation during the cellular differentiation process (See blue ovals on the figure).
