# Supplementary material for: A transversal approach to predict gene product networks from ontology-based similarity
Source: BMC Bioinformatics. 2007 Jul 2;8:235. doi: 10.1186/1471-2105-8-235 (PMC1940024; doi:10.1186/1471-2105-8-235)
Supplement: Additional file 4 — Selection of a similarity threshold for the Azuaje's approach. This file contains a graph representing the number of networks (red curve) and average number of gene products per network. (blue curve) according to each threshold. The combination of the criteria of selection, i.e. high degree of similarity and high number of gene products per networks, leads us to choose a threshold of .65 for the Azuaje's approach. [file 1471-2105-8-235-S4.pdf]

A transversal approach to predict gene product networks from ontology-based similarity

Julie Chabalier, Jean Mosser and Anita Burgun

Supplementary information: Selection of a similarity threshold for the Azuaje's approach

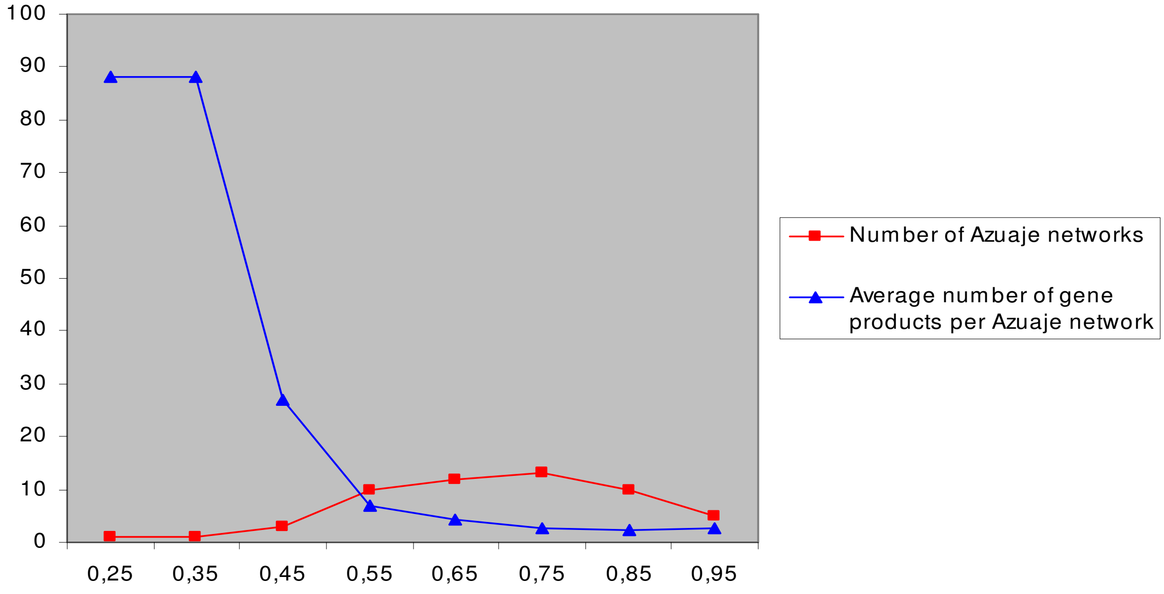

Number of networks (red curve) and average number of gene products per network. (blue curve) according to each threshold. The combination of the criteria of selection, i.e. high degree of similarity and high number of gene products per networks, leads us to choose a threshold of .65 for the Azuaje's approach.
