# Supplementary material for: A transversal approach to predict gene product networks from ontology-based similarity
Source: BMC Bioinformatics. 2007 Jul 2;8:235. doi: 10.1186/1471-2105-8-235 (PMC1940024; doi:10.1186/1471-2105-8-235)
Supplement: Additional file 5 — Azuaje comparison. This file contains a table presenting the networks obtained from the Azuaje methodology associated with their corresponding transversal networks (or part of networks) and their GO profiles. [file 1471-2105-8-235-S5.pdf]

A transversal approach to predict gene product networks from ontology-based similarity

Julie Chabalier, Jean Mosser and Anita Burgun

Supplementary information: Azuaje comparison

Grey rows represent identical networks

Azuaje network	*Gene product*	*Transversal result*	*GO profile*
1	RPL7A-RPL39-RPL41 RPS3RPS7-EIF4A2-EIF3S2-EIF3S8-ALG8-MAN2A1-HMGCS1-UGT2B17-AKR1C3-ACAS2	Partial Network 1<br>Partial Network 3	Protein metabolism<br>Cellular Biosynthesis<br>Lipid metabolism
2	GATM-ODC1-SMS-SEPHS2	Partial Network 2	Amine metabolism
3	ATP7B-SLC11A2	Partial Network 6	Ion transport
4	RNASE4 – SF3B2	Partial Network 9	RNA metabolism
5	SLC2A5 – SLC2A3	Network 15	Carbohydrate transport
6	NME2 – NME1	Network 11	Nucleotide metabolism
7	G22P1- HMGB1 – HMGA2 – PIAS4 – Q96B01	Partial Network 4<br>+ Q96B01	DNA metabolism
8	GSN – KRT8 – TUBA1	Network 10<br>+ GSN	Organelle organization and biogenesis
9	GAPD-UBE2D1- MBTPS1	Partial Network 5<br>Partial Network 3	Carbohydrate metabolism<br>Lipid metabolism<br>Cellular catabolism
10	H2AFY – NAP1L4 – NAP1L1<br>VAPB	Partial Network 4<br>Partial Network 7	DNA metabolism<br>Protein metabolism
11	TTR - CDH17- SLC35B1 – ARPC1B – CLDN1 – H3F3B-VTN – AFP - APOH	Network 18<br>Partial Network 4<br>+ TTR - CDH17-SLC35B1 –ARPC1B – CLDN1 –APOH	Defense response<br>DNA metabolism
12	TNFRSF10B – MTCH1	none	none
